# Automated fractal analysis of trabecular bone in multiple myeloma: a macro-based radiographic study

**DOI:** 10.1186/s12903-026-09300-8

**Published:** 2026-07-20

**Authors:** Ozlem Busra Dogan, Hatice Boyacioglu Erden

**Affiliations:** 1 Department of Dentomaxillofacial Radiology, Faculty of Dentistry, Bursa Uludag University, Gorukle, Bursa 16120 Turkey; 2 Department of Dentomaxillofacial Radiology, Faculty of Dentistry, Hacettepe University, Sihhiye, Ankara, 06230 Turkey

**Keywords:** Automation, Bone density, Fractals, Image processing, Mandible, Panoramic radiography

## Abstract

**Objective:**

This study aimed to investigate the applicability of fractal analysis for evaluating trabecular bone microarchitecture in multiple myeloma patients while implementing an automated macro-based workflow to provide reproducible and standardized post-processing analysis with reduced operator-dependent variability.

**Methodology:**

The study included 41 patients with multiple myeloma and 41 age- and sex-matched healthy controls. Fractal dimension measurements were initially performed manually using the conventional ImageJ/FracLac box-counting method. Subsequently, a macro-based automated workflow was evaluated for reproducibility and standardization of the analysis process.

**Results:**

No statistically significant differences were observed between the multiple myeloma and control groups for ROI1 (1.5329 ± 0.043 vs. 1.5368 ± 0.040, *p* = 0.674), ROI2 (1.5320 ± 0.042 vs. 1.5322 ± 0.041, *p* = 0.979), or ROI3 (1.5361 ± 0.049 vs. 1.5371 ± 0.048, *p* = 0.928). Intra-observer reliability was excellent (ICC = 0.91, 95% CI: 0.857–0.949), and agreement between manual and automated fractal dimension measurements was perfect.

**Conclusions:**

Fractal dimension analysis did not show statistically significant differences between multiple myeloma patients and controls in this cohort, suggesting limited discriminative value of the method for structural assessment of multiple myeloma under the present conditions. However, the macro-focused workflow was compatible with manual measurements and may provide a standardized and repeatable framework that could help reduce operator-induced variability in image processing and analysis, thereby supporting methodological consistency in future imaging research.

**Supplementary Information:**

The online version contains supplementary material available at https://doi.org/10.1186/s12903-026-09300-8.

## Introduction

Multiple myeloma (MM), a hematological malignancy characterized by the neoplastic proliferation of monoclonal plasma cells, systemically compromises bone integrity. This malignant expansion in the bone marrow leads to systemic symptoms such as anemia, infections, hypercalcemia, and renal failure. When myeloma cells invade the bone marrow, they inhibit osteoblasts, which are responsible for bone formation, and enhance osteoclast activity, which breaks down the existing bone. MM most commonly affects the jawbones, ribs, pelvis, and spine. Osteolytic lesions associated with MM cause bone structure deterioration and, particularly in the jawbones, microstructural changes [1, 2]. Given the high incidence of jawbone involvement (up to 35% in the literature) and the routine use of panoramic imaging in dental evaluations, radiologic assessment becomes essential in MM management [3]. Since these osseous changes may manifest as alterations in trabecular bone microarchitecture, quantitative image analysis methods such as fractal dimension (FD) measurement may provide an objective approach for their assessment and thereby support the rationale for the present study. Bone marrow transplantation is a common treatment for multiple myeloma (MM). Additionally, a comprehensive dental care program is recommended before initiating bisphosphonate (BP) therapy in MM patients to reduce the risk of drug-induced osteonecrosis of the jaw (MRONJ). These patients should be referred for a dental checkup to eliminate potential sources of infection [4]. For radiographic assessment of these patients, panoramic imaging represents a two-dimensional digital method offering a wide-angle view of the maxilla, mandible, teeth, maxillary sinuses, and temporomandibular joints. This modality offers various advantages, including low radiation dose, broad anatomical coverage, rapid and easy application, and widespread use in routine dental examinations.

To quantitatively assess bone microarchitecture on these radiographs, fractal analysis quantitatively characterizes complex bone pattern. Fractal analysis measures the self-similarity of complex and irregular structures. It is used in radiology, particularly to assess the complexity and density of trabecular bone structure. Fractal dimension (FD) quantifies structural complexity: higher values indicate greater density and hardness in the trabecular architecture, while lower values suggest a loss of density, structural deterioration, or degenerative changes in the bone tissue [5, 6].Various methods have been developed for calculating FD; among these, the box-counting method defined by White and Rudolph [7] is one of the most frequently used approaches. This technique allows for detailed analysis of the microstructural properties of trabecular bone and bone marrow microstructure.

The application of fractal analysis to panoramic radiographs offers diagnostic potential despite the resolution limitation of the panoramic technique [2]. Particularly in systemic diseases such as MM, panoramic radiography is among the methods providing diagnostic support. In multiple myeloma, trabecular bone disruption has been suggested to be associated with lower fractal dimension values, potentially reflecting alterations in bone microarchitecture. This quantitative capability makes panoramic radiography a valuable diagnostic tool in MM assessment. A review of the current literature indicates that only a limited number of studies have applied fractal analysis to panoramic radiographs to evaluate microstructural changes in the jawbones of MM patients [8, 9]. These studies investigated how MM-associated trabecular bone alterations affect FD values. Michels et al., [9] found no significant differences, Akyol et al., [8] reported lower fractal dimension values in affected patients. These heterogeneous findings highlight the need for further investigation to better understand the impact of multiple myeloma on jawbone microarchitecture. Given that dental professionals routinely evaluate such patients prior to transplantation, clarifying potential radiographic alterations may have clinical relevance for imaging-based assessment and monitoring.

Fractal analysis in ImageJ involves numerous sequential processing steps, such as ROI selection, image transformation, blurring, subtraction, thresholding, conversion to a mask, and Fractal Box Count, making it a highly time-consuming and user-dependent process when performed manually [7, 10, 11]. Manual completion of each step is labor-intensive, particularly in studies analyzing numerous images. This approach compromises reproducibility and introduces observer error through procedural omissions such as skipping steps [11, 12]. Therefore, automating the analysis process or standardising it using macro scripts enhances both efficiency and reproducibility in terms of time savings and more reliable results. Although several studies have proposed automated or semi-automated approaches for fractal analysis, including Python-based implementations [13] and general Fiji/ImageJ workflows [12], their application in dental panoramic radiography remains limited. In this context, the present study introduces a standardized macro-based workflow adapted for panoramic radiographic images, aiming to support consistent and reproducible application of the fractal analysis procedure within a dental imaging setting.

The aim of this study was twofold. First, to investigate whether fractal dimension (FD) analysis of panoramic radiographs could detect differences in trabecular bone microarchitecture between patients with multiple myeloma (MM) and healthy controls under standardized analytical conditions. Second, to develop and implement a macro-based workflow in Fiji/ImageJ to systematize the image processing and FD calculation steps, thereby enhancing methodological consistency and reducing operator-dependent variability in fractal analysis.

Given the known variability in manually executed FD workflows, this study sought not only to explore the potential structural characteristics of MM-related bone changes in the jaw, but also to examine whether a standardized analytical framework could provide reproducible and stable measurements suitable for comparative research.

## Materials and methods

This retrospective study conducted at the Faculty of Dentistry of Hacettepe University was performed in accordance with the applicable ethical principles, including the 1964 Declaration of Helsinki and its later amendments. The study was reviewed and approved by the Local Ethics Committee of Hacettepe University (Approval No: SBA 25/696). Due to the retrospective nature of the study and the use of anonymized data, the requirement for informed consent (consent to participate) was waived by the committee.

In accordance with the study’s retrospective nature, no additional study-specific informed consent form was obtained. However, a general written consent form regarding the use of patient data and images for scientific purposes is routinely obtained from all individuals upon admission to our clinics.

The inclusion criteria for all participants were: (1) being over 18 years of age and (2) having a panoramic radiograph of sufficient diagnostic quality in the digital archive. For the study group, a confirmed diagnosis of MM was required and verified through review of hospital records. For the control group, an absence of any other systemic disease was confirmed based on hospital information system records, with no recorded diagnosis or reported history of systemic disease at the time of radiographic acquisition.

Exclusion criteria for both groups were: (1) history of maxillofacial surgery, trauma, or fractures; (2) history of systemic or metabolic bone diseases known to affect the jaw (e.g., osteoporosis, diabetes mellitus, other malignancies); (3) diagnosis of any temporomandibular joint (TMJ) disorders; (4) a documented history of bisphosphonate use; and (5) incomplete demographic or clinical data. The study population consisted of a multiple myeloma (MM) group and a systemically healthy control group. All eligibility assessments were performed based on existing medical records, without prospective clinical examination.

**Fig. 1:**
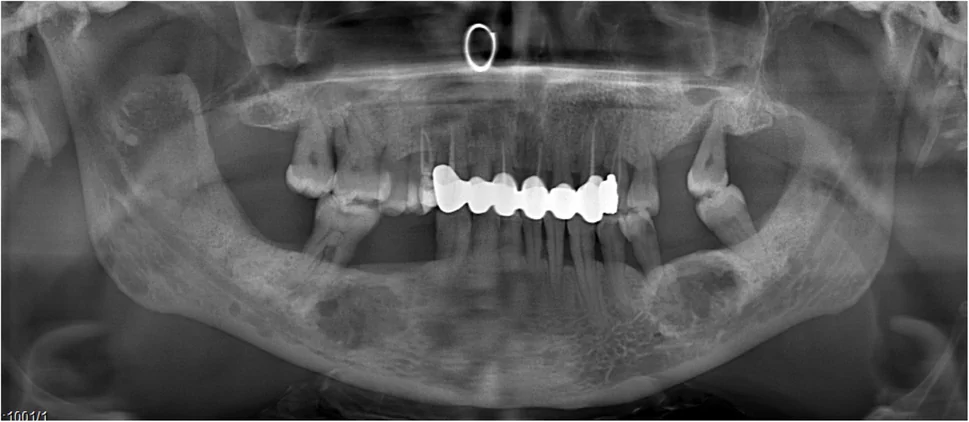
Panoramic image of a multiple myeloma patient excluded from the study due to the presence of a lytic lesion

Initially, the records of 54 patients with an MM diagnosis were reviewed. After applying the study criteria, 41 patients (15 females, 26 males; mean age: 64.59 ± 8.20 years) were included in the final study group. Eleven patients were excluded due to a history of bisphosphonate therapy, and two patients with multiple myeloma who presented with clearly visible lytic lesions were excluded, as advanced structural bone destruction was considered likely to influence trabecular fractal dimension measurements and potentially confound the analysis (Fig. 1). The control group comprised 41 systemically healthy individuals (15 females, 26 males; mean age: 64.59 ± 8.20 years) who were one-to-one age- and sex-matched with the MM group. Controls were selected from the institutional radiographic archive using a random sampling approach and applying the same inclusion and exclusion criteria as the MM group.

All panoramic radiographs included in the study were acquired using a Veraview IC5 digital panoramic unit (Morita Corporation, Japan). The exposure parameters were standardized, ranging from 60 to 70 kVp and 1–7.5 mA, with an exposure time of 5.5–10 s, in strict adherence to the manufacturer’s standard acquisition protocol. All images were exported in Tagged Image File Format (TIFF) for subsequent analysis. Image processing and analysis were performed using Fiji/ImageJ software (version 1.54p; National Institutes of Health, USA). Importantly, ROI selection was performed manually prior to the automated processing steps. To ensure standardization and reproducibility in analysis, this macro script captures the entire post-ROI processing workflow, ensuring that all steps are applied uniformly to various image sets with a single command. Any radiograph where the predefined regions of interest (ROIs) could not be reliably delineated due to anatomical superposition or significant artefacts was exclude from the analysis. All measurements were conducted by an oral and maxillofacial radiologist with 7 years of clinical experience. All measurements were performed on the same 15-inch laptop computer (Apple MacBook Air M3, Cupertino, California, USA) with a resolution of 2880 × 1864 pixels and 10-bit colour support to prevent differences arising from image resolution. ROI selection was performed by a single observer, as ROI placement followed standardized anatomical landmarks to ensure consistency. Care was taken to exclude any anatomical structures such as the lamina dura, periodontal ligament, alveolar crest, tooth root surface, mandibular canal, and mandibular base from the selected regions. To assess intraobserver agreement, 20% of the data was repeated 2 weeks after the initial evaluation was completed.

### Fractal analysis

**Fig. 2:**
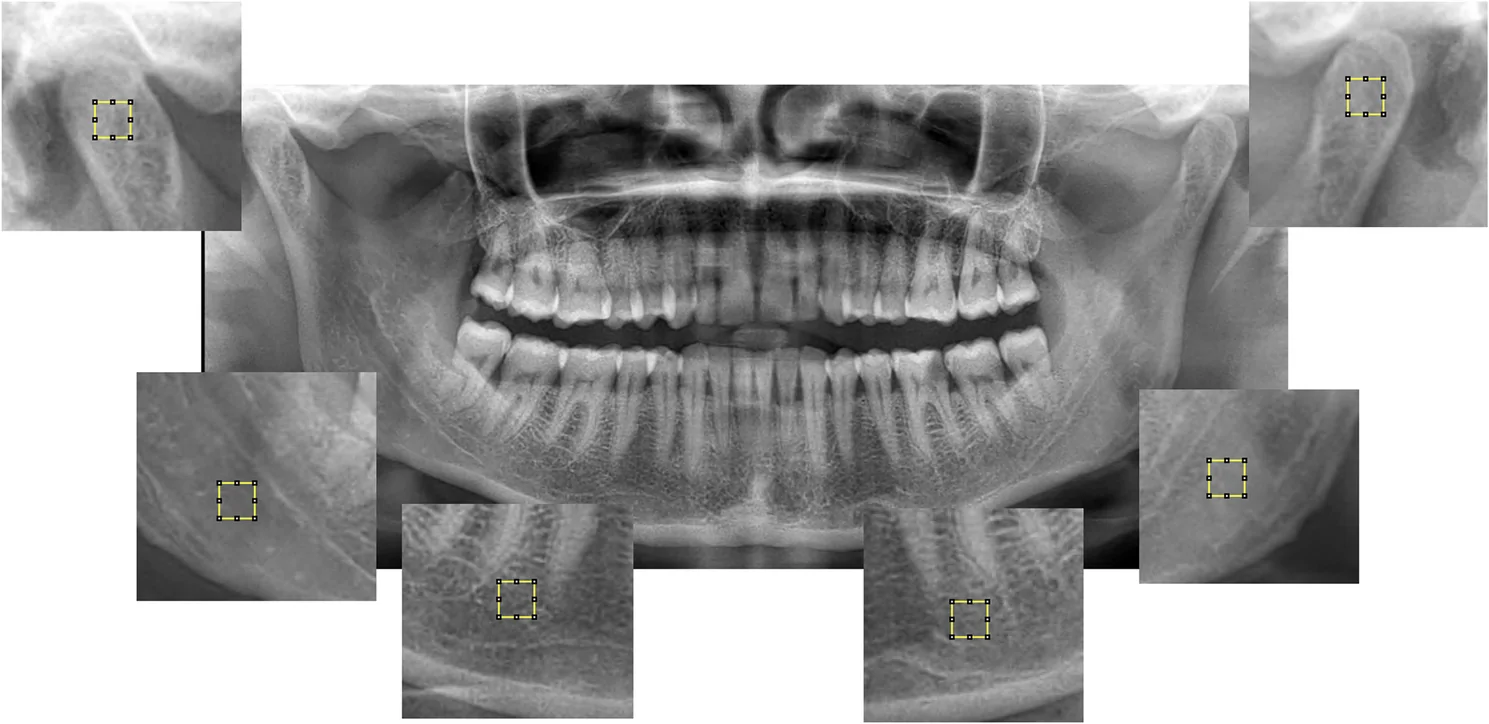
Selection of regions of interest (ROI) on panoramic radiographs. ROI 1: 60 × 60 pixel square at the mandibular condyle; ROI 2: 60 × 60 pixel square located inferior to the mandibular canal in the mandibular angle region; ROI 3: 60 × 60 pixel square located distal to the mental foramen in the mandibular body

The regions of interest (ROIs) were manually selected to ensure that no anatomical structures likely to influence the fractal analysis (FA) values were included within the selected areas. FD values were calculated as the mean of bilateral measurements from corresponding regions (Fig. 2).

Three 60 × 60 pixel ROIs were manually defined on each panoramic radiograph in the following trabecular bone locations:

1. ROI 1 (Condyle): Central trabecular bone within the mandibular condyle
2. ROI 2 (Angle): Trabecular bone inferior to the mandibular canal in the angle region
3. ROI 3 (Body): Trabecular bone distal to the mental foramen

The mandibular condyle (ROI 1) was selected due to its high remodeling activity and sensitivity to functional loading, making it potentially responsive to systemic bone metabolism alterations in multiple myeloma. The mandibular angle (ROI 2) and corpus (ROI 3) were included to represent regions with different biomechanical stress distributions, enabling regional comparison of trabecular bone changes.

**Fig. 3:**
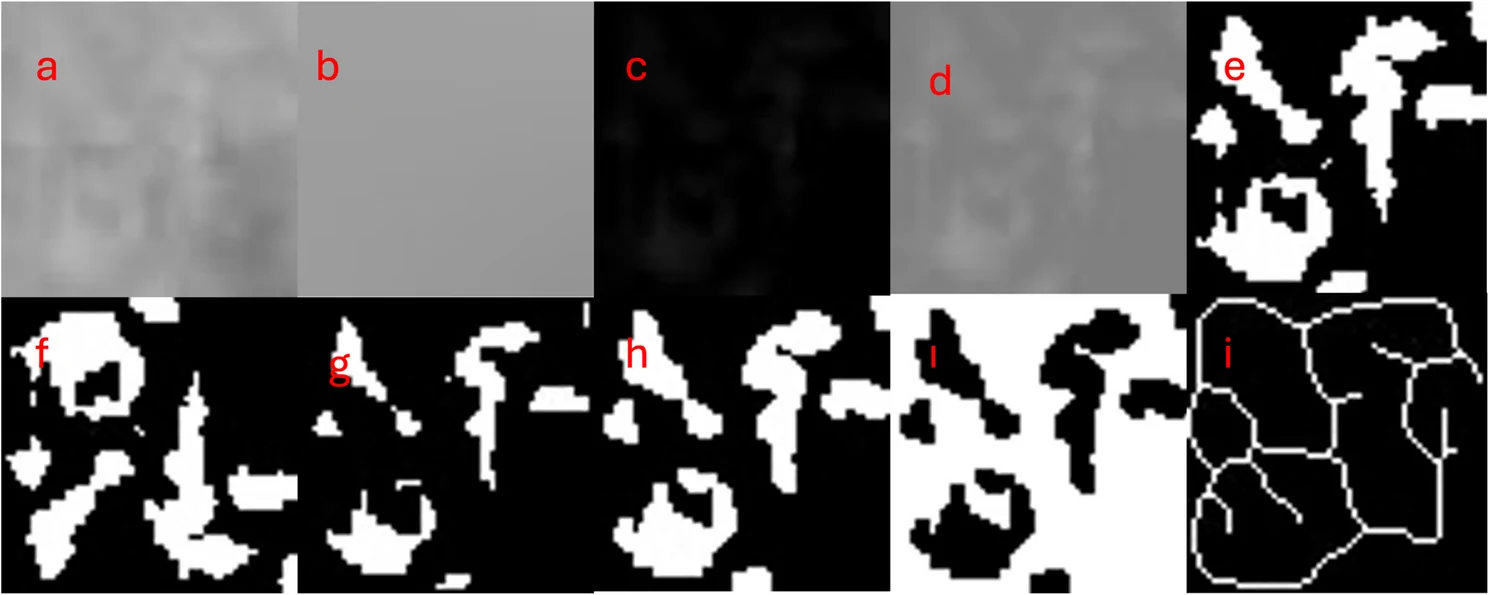
Image processing workflow for fractal analysis: **a** duplicated region of interest; **b** application of Gaussian blur filter; **c** subtraction of blurred image from original image; **d** addition of 128 grayscale; **e** thresholding; **f** binarization; **g** erosion; **h** dilation; **i** inversion; **j** skeletonization

Fractal analysis operations were performed by following the steps below (Fig. 3).

Duplicate: A duplicate of the original ROI was created to preserve the initial image.

Gaussian Blur Filter: A Gaussian blur (sigma = 35 pixels) was applied to reduce high-frequency noise. (White & Rudolph, 1999) [7]. In ImageJ’s Gaussian Blur filter, the kernel size is automatically calculated when you specify σ = 35. This ensures that the Gaussian distribution is adequately sampled without manual size input.

Subtraction: To isolate the trabecular structures, the blurred image was subtracted from the original image.

Add 128 Gray value: The image was subsequently normalized to an average gray value of 128. The images were first converted to 8-bit grayscale format in ImageJ to standardize pixel intensity values prior to further processing.

Thresholding: Automatic thresholding was applied to convert the images to binary format using the “Default” algorithm (Ridler & Calvard, 1978) [10], ensuring that the threshold was determined automatically. This method automatically determines an optimal threshold, providing consistent segmentation in images with heterogeneous contrast while preserving trabecular structures [11]. 

Skeletonization: Sequential morphological operations, including erosion, dilation, and inversion, were applied, followed by skeletonization to extract the trabecular structure of the bone.

Fractal Analysis: Box sizes followed a predefined geometric progression from 2 to 64 pixels. Linear regression was performed on the log-log plot, and FD was calculated from the slope (R² > 0.95 for all analyses) [7]. 

Following the completion of all macro tasks, the calculated fractal dimension (FD) values and corresponding messages were automatically displayed in the ImageJ log or results window. Using a custom macro script, we automated image processing steps based on the White and Rudolph [7] method, which were previously performed manually using Fiji (ImageJ, version 1.54p; National Institutes of Health, Bethesda, MD, USA). To ensure standardization and reproducibility in analysis, this macro script captures the entire processing workflow, ensuring that all steps are applied uniformly to various image sets with a single command. Manual quantitative analysis of these biomarkers require approximately 90 s per ROI including all processing steps. This automated workflow eliminated the need for manual processing, reducing analysis time to approximately 1 s per ROI.

### ImageJ macro implementation

Software Development: We developed a macro script for the ImageJ platform (version 1.54p; National Institutes of Health, Bethesda, MD, USA; accessible at http://imagej.nih.gov/ij) and implemented it using the ImageJ 1.x Macro language [12] in the Fiji image processing environment (http://fiji.sc/Fiji, public domain) in order to create an automated technique for fractal analysis in panoramic images.

User interface and macro execution were performed within the Fiji/ImageJ environment. The macro can be run directly in Fiji without installation; it may be opened either by drag-and-drop into the ImageJ window or via Plugins → Macros. Upon execution, the script proceeds through a predefined and standardized sequence of image processing steps to generate fractal dimension outputs.

Analysis Type: Users select which images will be analyzed. They have the option to record intermediate steps.Target directories for input images, output CSV files, and intermediate processing steps are user-definable.

Input Images: It is suggested that ROIs be manually drawn on panoramic images in order to achieve precise findings.

Customisable Parameters: Depending on their requirements, the user can change the parameter values at various process in the processing pipeline.

The automated macro required less than 1 second per ROI.

Validation Protocol: To assess accuracy, FD values from the automated macro were compared with manual measurements using 492 expert-selected ROI images (3 ROIs × 82 patients × 2 sides) from the study cohort.

Code Availability: The complete macro script is publicly available at https://github.com/doganob/imagej-fractal-analysis.

### Statistical analysis

An a priori power analysis (G*Power, version 3.1.9) determined that a minimum of 34 participants per group would be required to detect a medium-to-large effect size (Cohen's d = 0.8) with 80% power at α = 0.05 using an independent samples t-test. Following medical record review, 41 eligible patients were enrolled in the patient group, and 41 age- and sex-matched healthy individuals were recruited as controls. This sample size met the predefined criteria based on a priori power analysis and was considered to provide acceptable statistical power within the context of the study design.

Data analysis was performed using IBM SPSS Statistics Version 29. The normality of the data was assessed using the Shapiro-Wilk test, and the homogeneity of variances was assessed using the Levene test. Since all variables met the assumptions for parametric tests, parametric methods were selected.

Agreement between manual and automated macro script measurements was evaluated using intraclass correlation coefficient (ICC). Demographic comparisons between groups were performed using the independent samples t-test for age and the chi-square test for gender distribution. The main analysis included an independent samples t-test to compare fractal dimension values between multiple myeloma patients and the healthy control group. Data are presented as mean ± standard deviation. A *p*-value less than 0.05 was considered statistically significant. Intra-observer reliability was also assessed using ICC based on a randomly selected subset of ROIs to evaluate measurement consistency.

## Results

The study population consisted of 41 patients with multiple myeloma and 41 age- and sex-matched healthy controls. The mean age of the MM group and the control group was 64.59 ± 8.20. The male-to-female ratio was the same between groups; there were 26 males (63.4%) and 15 females (36.6%) in each group. As intended in the matching process, there was no significant difference between groups in terms of mean age (*p* = 1.000) or gender distribution (*p* = 1.000) (Table 1).

**Table 1:** Demographic and clinical characteristics of the study participants

Characteristic	Multiple Myeloma Group (*n* = 41)	Control Group (*n* = 41)	*p*-value
Age (years)			
Mean ± SD	64.59 ± 8.20	64.59 ± 8.20	1.000
Sex, *n* (%)			
Male	26 (63.4%)	26 (63.4%)	1.000
Female	15 (36.6%)	15 (36.6%)	

The control group was matched to the patient group for age and sex

*SD*  Standard Deviation

For each participant, bilateral measurements (from both the right and left sides) were taken from three distinct anatomical regions. This protocol resulted in six raw measurements per individual. Subsequently, for each of the three regions, the corresponding right and left-side values were averaged, yielding a single mean value per region per subject for final analysis. This approach is consistent with previous morphometric and radiographic studies, including Akyol et al., [8], where bilateral measurements were averaged to obtain a single regional value for statistical analysis.

The assumptions of normality (Shapiro–Wilk test, *p* > 0.05) and homogeneity of variances (Levene’s test, *p* > 0.05) were satisfied for all parameters. Three different fractal dimension (FD) parameters obtained from panoramic radiographs were compared between groups using independent samples t-tests. There were no statistically significant differences in the FD values of the MM group compared with the control group across ROI1, ROI2, and ROI3 regions (*p* > 0.05) (Table 2).

**Table 2:** Comparison of fractal analysis parameters between multiple myeloma patients and healthy controls

Parameter	Group	Mean (M)	SD	t	*p*	Cohen’s d
ROI1	MM	1.5329	0.043	-0.422	0.674	-0.093
	Control	1.5368	0.040			
ROI2	MM	1.5320	0.042	-0.027	0.979	-0.006
	Control	1.5322	0.041			
ROI3	MM	1.5361	0.049	-0.091	0.928	-0.020
	Control	1.5371	0.048			

*MM*  Multiple Myeloma, *ROI*  Region of Interest, *SD*  Standard Deviation

Intra-observer reliability was assessed at the ROI level. A randomly selected subset of 55 mean ROI values (representing approximately 20–25% of the total 246 mean ROI values) was re-evaluated. Each mean ROI value was calculated by averaging the measurements obtained from the right and left sides. The analysis demonstrated excellent intra-observer reliability. The ICC was calculated as 0.91 (95% CI: 0.857–0.949; *p* < 0.001), demonstrating statistically significant high consistency between repeated measurements.

The automated macro was tested on 492 ROI images from 82 participants (3 ROIs × 2 sides per patient). Agreement between automated and manual FD measurements was excellent (ICC = 1.00, 95% CI: 1.00–1.00, *p* < 0.05) reflecting complete computational equivalence between the macro-based output and the reference manual implementation of the fractal analysis procedure (Table 3). This result indicates deterministic reproducibility of the implemented workflow rather than variability arising from biological measurements.

**Table 3:** Agreement between automated and manual fractal dimension (FD) measurements

Parameter	Specification / Value
Sample Size	
- Patients	82
- Total ROI Images	492
- ROIs per Patient	3 ROIs × 2 sides
Agreement Analysis	
- Intraclass Correlation Coefficient (ICC)	1.00
− 95% Confidence Interval	1.00–1.00
- *p*-value	< 0.05
	Excellent agreement; identical values were obtained by both methods.

## Discussion

This study presents a macro-based implementation of a standardized fractal analysis workflow in Fiji/ImageJ. Rather than introducing a new algorithmic approach, the proposed method focuses on workflow automation of established image processing steps, aiming to standardize and streamline fractal analysis in panoramic radiographs and improve the reproducibility and consistency of measurements. The automated macro script demonstrated excellent reproducibility and perfect agreement with manual measurements. This supports the utility of the tool for standardized fractal analysis.Manual quantitative analysis of these biomarkers required approximately 90 s per ROI, including all preprocessing and fractal analysis steps. This time was recorded by an experienced oral and maxillofacial radiologist performing the full manual workflow under routine analysis conditions after ROI selection. In contrast, the automated workflow measured the time from initiation of the ImageJ macro to completion of fractal dimension output, following manual ROI selection, and reduced the processing time to approximately 1 s per ROI. Both measurements reflect practical workflow efficiency rather than experimentally controlled timing conditions. Additionally, the open-source availability of the macro may facilitate broader accessibility and independent evaluation. Fiji/ImageJ-based automated fractal analysis workflows specifically tailored for panoramic radiographs remain limited in the literature, suggesting that further methodological standardization may be beneficial in dental image analysis.

While the Python-based tool proposed by Tassoker et al., [13] provides automation for fractal analysis, its implementation requires familiarity with Python programming, which may represent a practical barrier for researchers primarily working within ImageJ-based environments. The macro presented in the current study was developed to facilitate integration within existing Fiji/ImageJ workflows, focusing on accessibility and procedural standardization rather than performance comparison. Although automated and semi-automated approaches have been described, including Python-based implementations [13] and general Fiji/ImageJ workflows [12], their direct adaptation to dental panoramic radiographs remains limited. In this context, the present study introduces a standardized macro-based workflow adapted for panoramic radiographic images, aiming to support consistent and reproducible application of fractal analysis within routine dental imaging practice.

Our findings demonstrated no significant differences in FD values between MM patients and healthy controls across all evaluated ROIs. In addition, the very small effect sizes observed across all ROIs suggest that, within the studied cohort, the biological impact of MM on mandibular trabecular complexity—at least as measured by FD on panoramic radiographs—may be limited. These findings contrast with Akyol et al., [8] who reported significantly lower FD in MM patients (*p* < 0.05), but aligns with Michels et al., [9] who also found no significant differences. Taken together, the available literature suggests heterogeneous results regarding the ability of FD analysis to detect trabecular alterations in MM.

Several factors may explain the discrepancy between these studies. First, differences in sample size alone do not appear to be the primary determining factor. Importantly, a key methodological distinction is that Akyol et al., [8] included multiple myeloma patients without bisphosphonate treatment (with a sample size of 67 patients per group)and with heterogeneous disease stages, incorporating patients at different stages of disease progression. This clinical heterogeneity in terms of disease burden and skeletal involvement may have increased the variability of trabecular structure within the MM group, potentially contributing to the statistically significant differences reported in that study.In contrast, Michels et al., [9] included a more structured cohort design, separating pre-treatment patients from those receiving intravenous bisphosphonate therapy (84 pre-treatment MM patients and 72 treated MM patients), in addition to 99 controls. Bisphosphonate therapy is known to influence bone remodeling and trabecular architecture, which may attenuate differences in fractal dimension measurements between groups.Furthermore, variation in disease stage and skeletal involvement across MM cohorts may further contribute to inconsistent FD outcomes, as trabecular complexity is highly sensitive to the severity and distribution of bone lesions.Collectively, these findings suggest that clinical heterogeneity—particularly disease stage and bisphosphonate exposure—may play a more important role than imaging methodology alone in contributing to the inconsistent results reported in FD-based assessments of multiple myeloma.

While the qualitative imaging characteristics of MM are well-documented, quantitative assessment of trabecular bone microarchitecture remains limited. Reducing observer-related variability in imaging assessment may contribute to more consistent evaluation of bone tissue characteristics and support objective quantitative analysis in research settings [14] Our findings in MM patients did not demonstrate statistically significant differences in trabecular bone microarchitectural parameters as assessed by automated fractal analysis. Given the visually apparent radiographic abnormalities observed in some multiple myeloma (MM) cases, the absence of significant differences in this study is noteworthy. The observed findings may be influenced by the fact that fractal dimension primarily reflects overall trabecular pattern complexity rather than isolated microstructural loss. In this context, previous studies have reported inconsistent results regarding FD changes in MM and related conditions.

While Michels et al., [9] and Feitosa et al., [15] reported no significant difference between groups using bisphosphonates for MM, Demiralp et al., [16] and Sevimay et al., [17] noticed more FD values in patients receiving bisphosphonates for various reasons. Given the potential influence of bisphosphonates on craniofacial trabecular bone complexity, individuals with a history of bisphosphonate therapy were excluded to minimize confounding effects and to better isolate disease-related changes. Notably, Feitosa et al., [15] also demonstrated a strong correlation between CBCT- and periapical-based FD measurements in MM patients.

Oravec et al., [18] used digital tomosynthesis in their study to analyze the thoracic and lumbar spine of MM patients and found that FD values increased in areas with vertebral fractures. The authors interpreted this finding within the context of spinal load-bearing bone, suggesting that FD changes may reflect structural remodeling associated with microfracture repair processes. However, this interpretation is specific to vertebral bone biomechanics and may not be directly transferable to the mandibular trabecular system due to anatomical and functional differences. Similarly, Takasu et al., [1] reported significantly reduced FD values in CT-based assessment of the L3 vertebra in MM patients, particularly in males. This finding supports the concept of trabecular disruption in weight-bearing skeletal sites; however, differences in imaging modality (CT vs. panoramic radiography) and anatomical region limit direct comparability with mandibular FD measurements. Therefore, although MM is biologically characterized by trabecular destruction that would theoretically result in reduced FD values, our findings did not demonstrate significant mandibular FD differences between groups. This apparent discrepancy may be related to anatomical site–specific remodeling patterns and the limited sensitivity of panoramic radiography in detecting subtle microarchitectural alterations.

Multiple factors may account for the inconsistencies reported across studies in the literature. Methodological differences between the studies in question may be the reason for these conflicting findings. FD values are highly sensitive to pre-processing steps such as the selected imaging method, ROI placement, image resolution, filtering, and binarisation. The lack of standardized image processing and ROI selection protocols among studies likely contributes to inconsistencies in reported FD values. The box-counting parameters (2–64 pixel sizes), and threshold algorithms also contribute to inter-study variability in FD calculations.

These findings suggest that while automated fractal analysis provides consistent, reproducible measurements, panoramic radiography may have limited sensitivity for detecting early MM-related trabecular bone changes in regions without visible lesions. Panoramic radiography has substantially lower spatial resolution compared to CBCT, potentially reducing sensitivity for subtle trabecular alterations. Future studies incorporating higher-resolution CBCT imaging and longitudinal assessment are needed to elucidate the utility of FD as a biomarker for progressive MM bone involvement. Recent longitudinal research evaluating fractal dimension together with panoramic radiomorphometric indices has demonstrated significant temporal changes in mandibular bone structure [19]. These findings underscore the importance of longitudinal designs, and further prospective studies are needed to better elucidate temporal alterations in fractal dimension across different patient populations. However, identifying subjects with accessible cone beam computed tomography scans poses methodological difficulties in retrospective analyses of specific cohorts. In prospectively designed studies, patient enrollment is frequently constrained by the low clinical presentation rate of individuals requiring cone beam computed tomography assessment.

Several limitations should be acknowledged.The exclusion of MM patients with overtly lytic lesions may have introduced a degree of selection bias and may limit the generalizability of the findings to cases with advanced radiographic bone destruction.Although the sample size met the a priori power analysis requirements, the relatively small cohort may still limit statistical power and increase the risk of a Type II error, and thus the negative findings should be interpreted with caution regarding robustness and generalizability. Due to the retrospective design and limited availability of detailed clinical and biochemical data, disease-related variables such as staging, duration, and laboratory parameters could not be incorporated into the analysis. This limits the ability to perform in-depth clinicoradiological correlation and to explore associations between trabecular bone microstructure and disease-related clinical parameters in multiple myeloma patients. The selection of regions of interest (ROIs) posed a challenge due to the heterogeneous radiographic features of multiple myeloma (MM), which can manifest differently even within the same patient. When evaluating the entire sample, minor positional adjustments were occasionally necessary during manual ROI placement within the same anatomical region in order to ensure inclusion of representative trabecular bone areas and to avoid cortical bone, tooth roots, and prominent ghost images inherent to panoramic radiographs. These adjustments did not involve changes in ROI size, which was consistently maintained at 60 × 60 pixels across all measurements. In this regard, manual ROI selection can be seen as both an advantage, due to its flexibility, and a disadvantage, as it carries the risk of observer-dependent variability. Our cross-sectional design precludes longitudinal evaluation of FD changes during treatment. Additionally, the retrospective nature of the study and the selection of control subjects based on existing hospital records may introduce a degree of selection bias. The exclusion of systemic conditions was also determined from medical records rather than prospective clinical or laboratory confirmation, which may limit the completeness of confounder control.

Panoramic radiography’s limited spatial resolution may reduce sensitivity for subtle microarchitectural changes. Although all panoramic radiographs were acquired using a single system with standardized exposure settings, minor variations within the preset ranges may still have influenced gray-level intensity and fractal dimension measurements. However, the use of a single device and a standardized protocol was intended to minimize technical variability across all images. Finally, our single-center design may limit generalizability. 

Future research should address key methodological limitations identified in the present study. (1) Automated ROI detection using deep learning could reduce operator dependency and improve reproducibility by eliminating variability associated with manual ROI placement. (2) Longitudinal assessment of FD changes during treatment may help overcome the limitation of the current cross-sectional design and evaluate the potential of FD as a monitoring biomarker. (3) Comparative studies between panoramic radiographs and CBCT could address limitations related to spatial resolution and improve validation against a more detailed imaging modality. (4) Validation of the macro script on multi-center cohorts is needed to address the single-center design limitation and to assess the generalizability and robustness of the proposed workflow across different imaging settings.

## Conclusion

This study presents that automated fractal analysis using a Fiji/ImageJ macro script provides standardized, reproducible measurements of mandibular trabecular bone microarchitecture in panoramic radiographs.

While no significant FD differences were detected between MM patients and controls in our cohort, the findings may be influenced by methodological and imaging-related factors, including limitations in panoramic radiography’s sensitivity. The validated automation tool addresses critical methodological gaps in fractal analysis workflows. The open-source availability and platform integration of the presented macro script facilitate widespread adoption and may enhance reproducibility in future studies investigating bone microarchitecture changes in MM.

## Supplementary Information

Supplementary Material 1.

## Data Availability

The data that support the findings of this study are available from the corresponding author upon reasonable request.

## Abbreviations

FD: Fractal dimension
ROI: Region of interest
ICC: Intraclass correlation coefficient
TIF: Tagged image file
MM: Multiple Myeloma
