# Supplementary material for: Automated fractal analysis of trabecular bone in multiple myeloma: a macro-based radiographic study
Source: BMC Oral Health. 2026 Jul 20;26:1692. doi: 10.1186/s12903-026-09300-8 (PMC13560388; doi:10.1186/s12903-026-09300-8)
Supplement: Supplementary file 1 [file 12903_2026_9300_MOESM1_ESM.docx]

MACRO SCRİPT

// --- 1) Assign Image Names to Variables ---

original_title = getTitle();

print("Main image to be analyzed: " + original_title);

// --- 2) Image Duplication ---

selectImage(original_title);

// Duplicate original image for reference

run("Duplicate...", "title=orig");

// Duplicate original image for preprocessing

run("Duplicate...", "title=prep");

// --- 3) Gaussian Blur ---

selectWindow("prep");

// Apply Gaussian Blur to remove low-frequency components

run("Gaussian Blur...", "sigma=35");

// --- 4) Subtract Blurred Image from Original ---

imageCalculator("Subtract create", "orig", "prep"); // original - blurred

// --- 5) Intensity Normalization ---

run("Add...", "value=128"); // Shift intensities to center around 128

run("8-bit"); // Convert to 8-bit grayscale

// --- 6) Thresholding ---

setAutoThreshold("Default dark no-reset"); // Apply Default threshold method

//setThreshold(128, 255);

setOption("BlackBackground", true);

run("Convert to Mask");

run("Erode");

run("Dilate");

run("Invert");

run("Skeletonize");

// --- 7) Fractal Analysis ---

run("Fractal Box Count...", "box=2,3,4,6,8,12,16,32,64 black"); // Box-counting method

// --- 8) Output of Results ---

print("--- Dynamic Analysis Completed ---");

print("Fractal Dimension (D) results have been displayed in the 'Results' window.");
